# Genomic Approaches for Improvement of Tropical Fruits: Fruit Quality, Shelf Life and Nutrient Content

**DOI:** 10.3390/genes12121881

**Published:** 2021-11-25

**Authors:** Malarvizhi Mathiazhagan, Bhavya Chidambara, Laxman R. Hunashikatti, Kundapura V. Ravishankar

**Affiliations:** Division of Basic Sciences, ICAR Indian Institute of Horticultural Research, Hessaraghatta Lake Post, Bengaluru 560089, India; malarvizhi.m@icar.gov.in (M.M.); bavyareddyc10@gmail.com (B.C.); laxman.rh@icar.gov.in (L.R.H.)

**Keywords:** tropical fruits, fruit quality, development, ripening, transcriptomics, metabolomics, SNP markers, QTL, CRISPR

## Abstract

The breeding of tropical fruit trees for improving fruit traits is complicated, due to the long juvenile phase, generation cycle, parthenocarpy, polyploidy, polyembryony, heterozygosity and biotic and abiotic factors, as well as a lack of good genomic resources. Many molecular techniques have recently evolved to assist and hasten conventional breeding efforts. Molecular markers linked to fruit development and fruit quality traits such as fruit shape, size, texture, aroma, peel and pulp colour were identified in tropical fruit crops, facilitating Marker-assisted breeding (MAB). An increase in the availability of genome sequences of tropical fruits further aided in the discovery of SNP variants/Indels, QTLs and genes that can ascertain the genetic determinants of fruit characters. Through multi-omics approaches such as genomics, transcriptomics, metabolomics and proteomics, the identification and quantification of transcripts, including non-coding RNAs, involved in sugar metabolism, fruit development and ripening, shelf life, and the biotic and abiotic stress that impacts fruit quality were made possible. Utilizing genomic assisted breeding methods such as genome wide association (GWAS), genomic selection (GS) and genetic modifications using CRISPR/Cas9 and transgenics has paved the way to studying gene function and developing cultivars with desirable fruit traits by overcoming long breeding cycles. Such comprehensive multi-omics approaches related to fruit characters in tropical fruits and their applications in breeding strategies and crop improvement are reviewed, discussed and presented here.

## 1. Introduction

Tropical fruits, grown mostly in low-income countries, are a treasure trove of nutrition and also assist in the economic sustainability of the region [1]. The rate of increase in global production of tropical fruits raised steadily from 5% to as high as 23% in 2019 and, based on the estimated projections, they will be one among the fastest growing agricultural sectors [2]. Tropical fruits possess innumerable health promoting bioactive compounds such as phenolic acids, carotenoids, flavonoids, anthocyanins, vitamins, minerals, fatty acids and fibre. Highly abundant antioxidants and phytochemicals present in these fruits make them an important nutritional source with good medicinal properties [3,4], and thereby help to achieve nutritional security for an ever-increasing world population.

Tropical fruits present a visual appeal to consumers through their attractive peel and pulp colour, unique aroma and taste. These attributes also directly denote the nutritional value of the fruits. Tropical fruits bear the brunt of unpredictable weather changes such as flooding, prolonged drought, pest and diseases, which are further aggravated by global warming [5]. Fruits need to be harvested at the right stages to have better marketability and prevent nutritional losses. Yet, because of their highly perishable nature, post-harvest losses are around 20–40% [6,7]. Hence, developing varieties with improved fruit quality, shelf life and tolerance towards abiotic and biotic factors becomes an essential component of breeding efforts.

**Figure 1:**
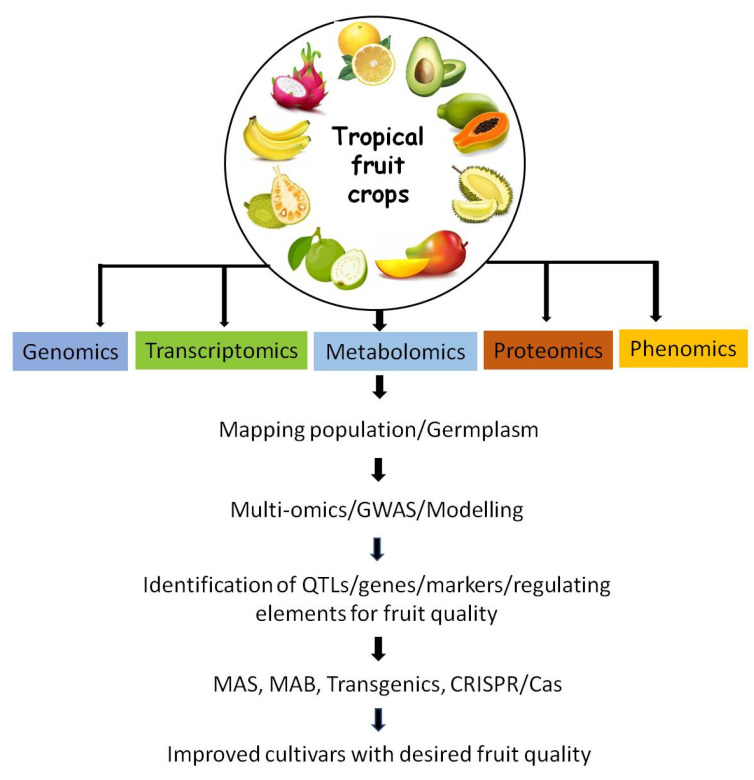
Genomic approaches in tropical fruit crops.

Tropical fruits with a longer shelf life are more suited for fresh consumption and processing. A long juvenile phase, irregular bearing, salinity, drought and wilt diseases all pose a major threat to fruit breeders and farmers. Achieving high yielding dwarf varieties with uniformly shaped fruit, good yield, desirable pulp, aroma, skin colour and uniform size with high nutritional content is desirable in the consumer market. Difficulties in conventional breeding can be minimized by employing molecular breeding techniques, supported by information on molecular markers, genetic linkage maps, whole genome sequence, genotyping by sequencing (GBS), transcriptome, metabolome and proteome profiles. This facilitates understanding of the functional roles of genes, transcription factors and pathways involved in fruit characters, ripening mechanism and resistance to disease and adverse environmental conditions [8,9]. Adapting an integrated ‘multi-omics’ approach aids in increasing the genomic knowledge and its applicability in developing improved cultivars (Figure 1).

Understanding fruit ripening and related traits, at the molecular and biochemical level, opened up possibilities for genetic modifications by the application of genetic engineering tools such as RNAi, CRISPR and antisense RNA technology to produce transgenic crops with enhanced fruit qualities and shelf life. Many proof of concept studies in model plants systems, such as for tomatoes and apples, have been developed which can pave the way for commercialisation of the transgenic varieties [10]. Information on the genomic approaches employed to study these intricate fruit ripening mechanisms and other fruit traits in some commercially important tropical fruits such as banana, mango, papaya, guava, citrus, jackfruit, dragon fruit, durian and avocado is discussed in this comprehensive review.

## 2. Fruit Development

Fruit development in climacteric and non-climacteric fruits is influenced by plant hormones and transcription factors. Fruit size, shape and weight are controlled by different genes, and their inconsistent expression results in physiological disorders. In mango, molecular markers like SSRs linked to fruit weight, width, volume, TSS, titrable acidity, ascorbic acid and total sugars, as well as reducing sugars, help in screening for varieties/seedlings with preferable fruit traits [11,12]. Spongy tissue is a serious physiological disorder affecting the edible quality of mango fruits, generating a metabolic profile containing stress related and flavour suppressing metabolites that differs among different stages of the fruit [13]. MAPKs are serine/threonine protein kinases involved in upstream and downstream regulation of signalling cascades by phosphorylation [14]. MAPK cascades are involved in plant fruit development via an ethylene signalling pathway [15]. A comprehensive transcriptome of banana reported that 10 *MAPKK* and 77 *MAPKKK* genes were involved in tissue development, fruit development and ripening and response to abiotic stress of cold, drought and salt [16]. Involvement of WRKY transcription factors during fruit development and biotic and abiotic responses in regulating mango malformation can help decipher the genetic control of the physiological disorder [17]. In banana, co-expression analysis of the B genome demonstrated the interaction of MbMADS75 with TFs such as bZIP involved in fruit development, ripening and stress responses [18].

Puffing disorder in citrus results in albedo breakdown and separation between peel and pulp. The transcript related to gibberellin and cytokinin signalling was repressed in affected albedo tissues, whereas expression of invertases and genes for sugar metabolism was upregulated in puffed fruits, suggesting their involvement in the disorder [19]. In papaya, the F_2_ mapping population was utilized to find QTLs that affect papaya fruit weight, diameter, length and shape. Six linkage groups (LGs) yielded fourteen QTL with phenotypic effects ranging from 5% to 23%, with clusters of two or more QTL on LGs 02, 03, 07,and 09. These genes were homologs of the tomato fruit QTLs ovate, sun and fw2.2, which control the size and shape of the fruit [20].

Comparative transcriptomics between guava leaf and fruits revealed a myriad of pathways involved in fruit development, such as ABA, ethylene, cytokinin, gibberellins (GA) and salicylic acid (SA) signal transduction pathways. Specifically, ethylene biosynthesis and signal transduction genes, 1-aminocyclopropane-1-carboxylate synthase (ACS), ACC oxidase 3, Ethylene receptor 2 (ETR2), ethylene response factor ERF-1, basic helix loop-helix (bHLH) TF and pyridoxine biosynthesis gene *PDX1.2*, were found upregulated during the development of fruit. Around 40 transcription factor families belonging to *MYB*, *MADS*, *HB*, *WRKY*, *ARF*, *bHLH*, *AP/EREBP*, *bZIP*, *NAC*, *AUX/IAA*, *B3*, Jumonji and Polycomb were found to be regulating the fruit development in guava [21]. The RAPD markers were widely used for molecular characterisation among different varieties of *Psidium* sp., and lately they were utilized for discriminating different fruit shapes such as ‘round’ and ‘pyriform’ using cluster analysis [22]. In jackfruit, accessions with fruit shapes viz. oblong, oval, ellipsoidal and clavate fruits with varying fruit weights and pulp colours were clustered separately using molecular markers [23,24]. These DNA markers can help in screening the seedlings for desired fruit shape.

## 3. Fruit Ripening

Fruit ripening is a complex process, during which flavour and colour development, cell wall modifications and softening, starch degradation and aroma development occurs, which are major factors contributing to the unique fruit characters. The process involves a complex network of the interaction between the plant hormones and critical transcription factors that regulate it at different levels. Different ‘omics’ approaches were employed to decipher the intricate mechanisms governing fruit ripening in tropical fruits.

### 3.1. Fruit Softening

When the fruit enters the ripening stage, there is a burst in ethylene production in climacteric fruits which accelerates the ripening process and brings about changes in cell wall architecture, fruit texture and, finally, firmness. Cell wall softening is an important physiological process during ripening, which is brought about by the solubilisation of pectin, depolymerisation of polysaccharides and modifications in cell wall components. Polygalacturonase (PG) plays an important role in ripening-associated pectin modifications, resulting in fruit softening [25]. In addition, other enzymes such as pectin methylesterase, (1→4)-β-glucanase and β-galactosidase also contribute to softening in tropical fruits [26]. Comparative transcriptome analysis of unripe and ripe fruits of banana, mango, guava and papaya exhibited differential expression of genes associated with fruit softening viz. pectin lyase (PL), xyloglucan endotransglucosylase/hydrolase protein 32 (XTH), polygalacturonase (PG), expansins and ethylene biosynthesis enzymes such as ACC synthase (ACS) and ACC oxidase genes [27,28,29,30,31]. Studies on peel during ripening in banana revealed the involvement of ERF and bHLH family TFs, auxin signalling related genes and xyloglucan endotransglucosylase/hydrolase (XTH) family members in peel softening [32]. Further, ethylene inducible, nuclear localised transcription factors involved in regulating fruit ripening were characterised by transcriptomic and gene expression studies in banana. This includes *MaMADS2*; *DREB* family genes; *TCP* family genes; and basic leucine zipper (*bZIP*), *ERF*, *NAC* and *C2H2* family genes [33]. The TFs *MaTCP5* and *MaTCP20* were known to act as transcriptional activators, whereas *MaTCP19* acted as a transcriptional inhibitor by directly binding to promoters of *MaXTH10/11* involved in fruit softening [34].

In addition, the interaction of banana TFs *MaBZR1/2* with *MaMPK14* promoted the transcriptional inhibition of cell wall modifying genes including *MaEXP2*, *MaPL2* and *MaXET5* [35]. Furthermore, *MaMYB3* negatively impacted starch degradation by directly repressing starch degradation-related genes, and thereby delaying banana fruit ripening [36]. Transcription factor *MabZIP93* positively regulated banana ripening by activating cell wall modifying genes such as *MaPL2*, *MaPE1*, *MaXTH23* and *MaXGT1* [37]. In contrast, *MabZIP74* negatively regulates the ethylene biosynthesis by repressing transcription of ethylene biosynthetic genes *MaACO1* and *MaACO4* [38] (Table 1).

Non-coding RNAs play a crucial role in most of the plant’s physiological conditions, including fruit ripening. In banana, out of 128 total miRNAs identified, 22 were differentially expressed in response to ethylenes that were known to target the genes encoding proteins such as GATA, ARF, DLC and AGO, etc. [82]. Long non-coding RNAs identified in banana had similarities with miRNAs involved in plant and seed development and biotic and abiotic stresses [83]. A study had also identified 46 and 25 miRNAs that are up and downregulated, respectively, during fruit ripening in banana. The main target genes of these miRNAs were found to be involved in miRNA biogenesis, fruit softening and aroma biosynthesis [84] (Table 2). The epigenetic regulation of fruit ripening genes has been reported in banana and papaya. Histone deacetylases (HDACs) are involved in deacetylation of major histone proteins. Seventeen MaHDACs were identified, among which MaHDA6 was found to control fruit ripening by histone deacetylation of the transcription factor genes, *MaERF11/15* [85]. Similarly, *CpHDA3* mediated the expression of pectin methylesterase and polygalacturonase genes during papaya fruit ripening and softening by forming a histone deacetylase repressor complex with *CpERF9* transcription factor [86].

Auxin/indole-3-acetic acid (Aux/IAA) family genes were also shown to be differentially regulated during banana and papaya ripening [32,87]. Several *CpARFs* and *CpEIL1* genes were involved in fruit ripening via the regulation of the auxin and ethylene signalling pathway [56,57]. Investigation of the ripe papaya transcriptome utilizing a cross-species microarray technique based on the evolutionary proximity of papaya and *Arabidopsis thaliana* resulted in the discovery of 414 ripening-related genes. Analysis revealed the regulatory control of transcription factors from MADS-box, NAC, GRF and AP2/ERF gene families in papaya ripening [53,88]. The specific binding of *CpMADS4* and *CpNAC3* to promoters of ethylene signal genes *CpERF9* and *CpEIL5* acts as a transcriptional activator in papaya ripening [54]. Similarly, TFs *CpMYB1* and *CpMYB2* had a negative regulatory role in ripening by suppressing the cell wall degradation genes such as *CpPME1*, *CpPME2* and *CpPG5* [55], whereas TFs *CpSPLs* guided by CpmiR156 were found to be involved in the ethylene signalling pathway [58] (Table 1).

Cultivars “Chanee” and “Monthong” are the two extensively studied commercial cultivars of durian. “Chanee” has a faster ripening process than “Monthong” and is validated through transcriptome and metabolome studies, which showed the over expression of DNA binding with one finger (Dof) transcription factors, auxin response factor (*DzARF2A*), ethylene and auxin resulting in the early onset of ripening in “Chanee” [79,81,89]. In addition, the role of ethylene response factor (ERF) transcription factors in ripening was annotated to be both positively and negatively correlated to ethylene and auxin mediated pathways [80,81] in durian fruit pulp.

Transcriptomics have helped decipher molecular mechanisms and networks governing fruit development and ripening in citrus species [90]. Late ripening traits in citrus were found to be regulated by TFs such as ERF1, ARF1 and TGA9 involved in ethylene, auxin and salicylic acid signal transduction pathways. Further, 9-cis-epoxycarotenoid dioxygenase (*NCED1*) was also found to play a critical role in late ripening [65]. Similar studies with a late ripening sweet orange mutant revealed 16 differentially expressed TFs during the ripening of WT and MT that were assigned to the families of C2H2, Dof, bHLH, ERF, MYB, NAC and LBD. The study identified that TF’s expression correlated to abscisic acid (ABA), citric acid, fructose, glucose and sucrose such as *RD26*, *NTT*, *GATA7* and *MYB21/62/77* [91]. In contrast, the early ripening trait was under the regulation of the MADS transcription factor in citrus [66], whereas in grapes, ROS and pathogenesis related genes were contributing to earliness [92]. In non-climacteric fruit crops such as citrus, ABA signalling combined with ethylene and jasmonic acid plays an important role in the ripening process. UPLC-Q-TOF-MS-based metabolomic profiling [93] and the phosphoproteomics of the chromoplast of sweet orange helped in identifying metabolites and phosphoproteins involved in fruit ripening, respectively, thereby revealing protein regulation via post-translational modifications in the chromoplast [94]. These studies further reiterate the fact that the fruit’s textural changes are brought about by the cell wall degrading enzymes, hormones, regulatory TFs and miRNAs, playing a crucial role in fruit softening, which is an important indicator of the fruit ripening phenomenon in tropical fruit crops.

**Table 2:** Non-coding RNAs identified in tropical fruits.

Crop	Source Seed	Non Coding RNAs Identified	Targets Identified	Response To	Reference
Mango	EST database of mango	miRNAs—3	94	Fruit development, ripening	[95]
	RNA-seq database	miRNAs—104 lncRNAs—7610	2347	Low temperature stress	[96]
	Genome assembly	tRNA—598 rRNA 45 snoRNA—47 snRNA—200 miRNA—235	—	-	[97]
	EST database of mango	miRNAs—18	44	Ripening	[98]
Banana	Transcriptome	miRNAs—59	120	Salt stress tolerance	[99]
	Transcriptome	miRNAs—82	815	Ripening	[100]
	Transcriptome	miRNAs—22	12	Ripening	[82]
	Transcriptome	miRNAs—46	944	Fruit softening and aroma biosynthesis	[84]
	Transcriptome	lncRNAs—12,462	—	Low temperature stress	[101]
Citrus	Transcriptome	miRNAs—101	28	Alkaline stress	[71]
Papaya	Transcriptome data	miRNAs—213	1741	Ripening	[102]
Guava	Guava genome, miRbase database	miRNAs—40	49	Salinity stress	[103]
Dragon fruit	Transcriptome data	lncRNAs—11,650	—	Betalain biosynthesis	[76]
	Genome assembly	miRNAs—4989 tRNAs—4857 rRNAs—5909 snRNAs—3877	—	Betalain biosynthesis	[74]

### 3.2. Sugar Metabolism

Fruit sweetness is an important characteristic feature determining fruit quality. During maturation and ripening of mango pulp, starch hydrolyses to sucrose, fructose and glucose with varied concentrations catalysed by enzymes such as invertases and β-glucosidases, giving a unique sweetness to the varieties [104]. In bananas, a total of 82 differentially expressed miRNAs were found to be targeting TFs and other functional proteins such as β-galactosidase, β-glucosidase, APETALA2, SPL, EIN3 and E3 ubiquitin ligase, involved in sugar metabolism during ripening [100]. In jackfruit, transcriptome analysis in different stages of perianth maturation and ripening has shed light on sugar metabolism. The stored starch gets converted to sucrose, thereby increasing the sweetness of the ripened fruit. Gene expression studies validated the upregulation of genes involved in starch degradation, sucrose synthesis, pectin, aromatic compounds and carotenoid synthesis during ripening [105]. The high throughput proteomics (LC–MS/MS) of slow and fast ripening avocado fruit revealed less abundant and higher abundant proteins in slow ripening phenotype, which might act as new markers for differential ripening in avocado [106]. A combined study of proteomics and metabolomics revealed that heat treatment will induce the ripening homogeneity in avocado by enhancing the accumulation of soluble sugars such as sucrose and galactose and other stress related enzymes [107]. Harvesting durian fruits at the correct maturing stage is a crucial factor for its marketability. It can be accomplished by employing a non-destructive method of analysing peduncle liquid for the presence of markers such as sucrose, asparagine, arginine and pipecolic acid for estimating the right stage for harvest [108]. In citrus, TFs such as *MYB21* along with C2H2, Dof family and some GATA family were found to be regulating the sugar metabolism and quinic acid, and citric acid production [91]. Further, five sucrose synthases (SUSs) and ten invertases (INVs) were established to be involved in sucrose degradation into arabinose, xylose and hexose in *Citrus maxima* “seedless” varieties [109].

### 3.3. Flavour

Flavour development in fruits increases at the onset of ripening, and a complex mixture of volatile metabolites such as aldehydes, alcohols, furanones, esters, ketones, lactones, terpenoids, etc., contribute towards the unique aroma of tropical fruits. Flavour development was also dependent on the cultivar and maturity stage of the fruit. Different volatile compounds, in particular α-Pinene, were found to be involved in flavour development during different stages of ripening in mango [110,111,112]. They can also serve as biomarkers for identifying fruit maturity and varieties, as these volatiles that contribute to the fruit aroma were specific to the unripe and ripe stage of the fruit [113]. There has been a complex regulation of the transcriptional process linked with aroma development during banana fruit ripening. Studies reported a few aroma biosynthetic genes such as *MaBCAT1*, *MaACY1*, *MaOMT1*, *MaMT1*, *MaGT1*, *MaAGT1* and *BanAAT* to be under the transcriptional control of TFs, *MabZIP4* and *MabZIP5* in banana [42]. During banana fruit ripening, the expansin and xyloglucan transglycosylase/hydrolase (XTH) families of genes that were related to cell wall degradation, synthesis of aromatic volatiles, ethylene biosynthesis, perception and signalling were found to be upregulated [15]. Ripe banana pulp contained higher fold expression of alcohol dehydrogenase (ADH), lipoxygenases (LOX) and transferases such as benzoyl transferases, methyltransferases and acyltransferases which have a potential role in aroma development [30]. TFs *MaDof10*, *23*, *24* and *25* were found regulating 10 ripening-related genes which are associated with cell wall degradation and aroma formation in banana fruits [40].

Comparative proteomic analysis of exocarp in papaya identified a total of 3220 proteins with significantly altered flavonoid and fatty acid metabolisms which may be associated with papaya fruit’s volatile formation [114]. Stage-specific metabolites during fruit development and ripening in papaya were identified which can serve as biomarkers and help unravel the aroma development in fruits. *CpLIS1*, *CpP450-2*, *CpAAT1* and *CpACX1* genes were found to be involved in the synthesis of volatiles’ compounds such as linalool biosynthesis, linalool oxygenases, esters or lactones during fruit ripening in papaya [115]. Aroma profiling of immature, mature green and mature yellow guava fruit detected volatiles such as (Z)-3-hexenyl acetate, ethyl butanoate and ethyl octanoate in abundance only in the mature yellow stage of fruit development. Interestingly, volatile compounds such as limonene, (E)-cadina-1,4-diene and β-ionone were accession specific with the potential to be used as biomarkers [64]. Furthermore, chromosome level assembly of the dragon fruit genome reported Orthologs Gene Clusters(OGCs) that were expanded in dragon fruit, contributing to fruit ripening and its unique flavour [116].

Durian fruits have a distinct flavour and unique taste which determine the commercial value of the fruit. Cultivar specific metabolome analysis associated with fruit odor and ripening process paved the way for a better understanding of those traits. Flavour related amino acids (cysteine, leucine) and organic acids along with sulphur containing γ-glutamylcysteine and glutathione contribute to the unique odor and taste of durian fruits [90]. To support this claim, expression of the leucyl aminopeptidase gene (*DzLAP1*) involved in glutathione metabolism was found upregulated during the unripe, mid-ripe and ripe stages of durian pulp [117]. Furthermore, a combined transcriptomics and metabolomic analysis of the ripening process provided a detailed account on metabolites, genes and pathways responsible for sour and umami taste and sulfuryl and fruity aroma of durian fruits during unripe and ripe stages of fruit development [118]. Interestingly, identification of ethionine as a precursor for odor inducing ethanethiol in durian pulp has opened up new insights into aroma research in durian fruits [119].

### 3.4. Pulp and Peel Colour

The colour of the pulp and peel is due to the presence of carotenoids and anthocyanins in higher concentrations and the fast degradation of chlorophyll. The accumulation of pigments in mango pulp and peel differs among cultivars. Genes involved in the carotenoid biosynthesis pathway such as *CRTISO*, *PDS*, *PSY*, *ZDS*, *LCYb*, *LCYe*, *BCH* and *ZEP* were found to have a positive correlation with total carotenoid content in pulp [120,121], whereas peel colour is found to be influenced by the expression of phenylalanine ammonia lyase (PAL) and p-coumarate 3-hydroxylase (C3H), key enzymes in anthocyanin biosynthesis [122]. Varieties with red peel had increased expression of anthocyanin biosynthesis genes compared to yellow and green peeled cultivars, whereas yellow peeled cultivars had higher fold of expression of genes associated with carotenoid biosynthesis than was observed in mango [123,124]. The amount of anthocyanin and bioactive compounds such as phenolic acids, antioxidants and carotenoids in mango peel was higher compared to pulp, which correlated with the abundance of transcripts related to the anthocyanin pathway in peel [125,126]. Further, transcriptome studies showed that bagging and shade conditions influence the peel colour, with reduced red coloration [127] (Table 3).

In banana, the RNA sequencing of epidermal cells of the purple and green parts of peel suggested that anthocyanin gene expression and, subsequently, anthocyanin metabolism contributed to the formation of the purple peel of “Minhou”, a wild banana [128]. Carotenoids accumulated during banana ripening due to the high expression of genes such as *LCYβ* (Lycopene β cyclase) and *GGPS* (Geranylgeranyl diphosphate synthase), especially *MaLCYB1.1* and *MaLCYB1.2*, regulated by ripening inducible transcription factor, *MaSPL16* and low expression of *CCD*s (Carotenoid cleavage dioxygenase 1) and *NCED*s (9-cis epoxy carotenoid dioxygenase) in ripe banana [30,46]. The peel color of citrus fruit changes from green to yellow or orange during fruit maturation. The green color is derived from chlorophyll in chloroplast, and chloroplast changes to chromoplast, accumulating carotenoids during fruit maturation. Integrated transcriptome and proteome study in red-flesh sweet orange “Cara Cara” and a yellow fleshed cultivar “Newhall” identified thirteen differential expressed genes involved in carotenoid metabolism, especially, carotenoid cleavage dioxygenases contributing to red colouration in pulp and peel [67,129]. Orange pericarp mutants were also used to study the peel colouration in citrus, which accumulated more β-carotene than the wild type [130] and suggested the involvement of TF and CcGCC1 (GARP and coiled-coil) expression with peel ripening [131].

The yellow peel colour in papaya is because of the accumulation of carotenoids such as lutein and β-carotene. The expression profile of genes involved in the metabolism of carbohydrate, chlorophyll and carotenoid revealed the mechanism of pulp softening and coloration of papaya. Inhibition of chlorophyll biosynthesis along with α-branch of carotenoid metabolism (*CHYB*) was found to be responsible for the yellow colour of papaya pulp, while both α- and β-branch of carotenoid biosynthesis contributed to yellow peel colour in papaya (Table 3). The papaya fruit transcriptome was enriched with genes that encode enzymes in the carotenoid biosynthetic pathway (*PSY1*, *PSY2*, *PDS1*, *PDS2*, *ZDS*, *CYCB*, *LCYB1*, *LCYB2*, *LCYE*, *CHYB*, *LUT1*, *VDE*, *ZEP*), among which *CpPDS* and *CpZDS* were more highly expressed in maturing fruits and red-fleshed fruits than in yellow-fleshed papaya fruits [132,133]. Further, transcription factors *cpNAC1*, *cpMYB1*, *cpMYB2*, *CpbHLH1*, *CpbHLH2* and *CpEIN3a* were found to regulate carotenoid biosynthesis by binding to *cpPDS2/4*, *CpZDS*, *CpLCY-e*, *CpCYC-B*, *CpLCY-B* and *CpCHY-b* genes [55,59,60,61]. Similarly, among three different coloured pulps of papaya, yellow pulp cultivars had high expression of *CpLCY-β2* and *CpCHX-β* genes with high accumulation of yellow pigments such as β-cryptoxanthin, zeaxanthin and violaxanthin, followed by orange pulp and, then, the red pulp [134].

Comparative RNA sequencing analysis between varieties with different peel colour and pulp colour revealed sixty-eight genes that may be involved in pulp and peel colour in guava, belonging to pathways such as ethylene, ABA and secondary metabolite biosynthesis. Transcription factor families of *WRKY*, *AP2*, *bHLH*, *PHOR1* (ubiquitin ligase activity), *MYB* and *C2C2.CO* were found likely to be involved in fruit ripening. Reticulin o-methyltransferase was found to contribute to red colouration in peel and pulp tissues. Candidate genes whose expression correlated with red peel colour were glycerol-3-phosphate acyltransferase 5 (*GPAT5*), peamaclein, CTP synthase-like, chloroplastic monodehydroascorbate (*MDA*), probable 2-oxoglutarate dependent dioxygenase AOP1 (*2OG-AOP1*), methionine synthase (*MS*), Secoisolariciresinol dehydrogenase (*SDH*) and BEL1-like homeodomain 1(*BLH1*) [21]. In *Psidium cattleyanum*, where red and yellow fruits are common, the peel transcriptome revealed the involvement of anthocyanidin synthase (*ANS*) and UDP-glucose: flavonoid-o-glucosyl transferase (*UFGT*) in red peeled fruits [135] (Table 3).

Betalain production in dragon fruit contributes to the nutritional and visual appeal of the fruit. The peel and pulp colours of the fruits were governed by the betalain pigments. Regulation of betalain production and sugar accumulation is found to be controlled by WRKY transcription factors *HpWRKY44* and *HpWRKY3*, respectively, by transcriptional control of cytochrome P450-like (HpCytP450-like1) and sucrose metabolic genes in red dragon fruit [75,136]. A combined transcriptomic cum metabolomic analysis further provided strong evidence that WRKY is involved in upregulation of cytochrome P450 enzymes (CYP76Ads), and it is the crucial step determining the red or white pulp of dragon fruits [137]. In addition, through comparative transcriptome analysis, *HpCYP76AD4*, *HpDODA1*, *HpDODA2* and *HpCYP76AD4* genes were found to contribute to the betalain/red pulp formation regulated by *HpNAC*, *HpGSTs* and MYB transcription factors [76,77]. A combined RNA sequence and proteomic analysis of red and white pulp fruits deciphered the molecular and post transcriptional level mechanism in betalain biosynthesis [138].

The metabolic profiling of different dragon fruits has enabled the classification of accessions based on fruit size rather than fruit pulp colour [139], and also demonstrated that the red peel of *Hylocereus polyrhizus* contained higher amounts of metabolites compared to the green peels of *Hylocereus undatus*, signifying the nutritional benefits of dragon fruit peels [140]. Fruit development stages in dragon fruit exhibited signature metabolites that are stage specific and tissue specific which can serve as biomarkers for quality assessment. Citramalic acid was found predominantly in unripe pulp, whereas malic acid and sugars dominated at the ripe stage. In peel, betaxanthin content was higher compared to betacyanin in different stages and vice versa in pulp [141]. In contrary, RNA sequence analysis revealed that red pulped *H. polyrhizus* saw a surge in genes involved in betacyanin and betaxanthin synthesis at all stages of pulp coloration, whereas white pulped *H. undatus* did not show any significant change [138]. Recent studies had also emphasised and debated the co-occurrence of anthocyanins along with betalains in red pulped dragon fruits [137,142]. The studies reveal that the color breaking step of peel and pulp in fruits is strongly under the regulation of TFs which control the carotenoid, anthocyanin and betalain biosynthesis pathway genes.

### 3.5. Fruit Quality

In fruits, sugars and organic acids, the sugar/acid ratio plays a more specific and important role in contributing to their unique taste and quality. SRAP markers specific to vitamin C content (VCC) were found very informative in guava landraces with high and low VCC. These markers have the potential to be incorporated in breeding to screen varieties with high VCC in graft or seedling stages [146]. In banana, the TF *MaSPL16* stimulated the transcription of lycopene β-cyclase genes and acts as an activator to modulate banana carotenoid biosynthesis [46]. Further, carotenoid cleavage dioxygenase 4 (*CCD4*) was found to be contributing to low carotenoids in Cavendish cultivars, whereas conversion of amyloplasts to chromoplasts was attributed towards high carotenoids in Asupina cultivars [147]. Similarly, the expression levels of genes involved in carotenoid biosynthesis in durian have been characterised, which can provide a boost in breeding for fruit nutritional quality [148].

Transcriptomics combined with network analysis helps in identification of regulatory mechanisms involved in acid accumulation and citrate in sweet orange cultivars [149]. Gene expression analysis in lemon genotypes with varying acidity facilitated the identification of genes contributing to the sweet or acidic taste. Putative genes such as citrate synthase, malate dehydrogenase, proton-pumping ATPase and flavanone 3-hydroxylase were positively correlated with acidity in citrus [150]. Moreover, natural variations in the promoter region of flavanone 3-hydroxylase (*CitF3H*) contributed to a change in dihydrokaempferol levels, leading to alteration in flavonoid content in different progenies in citrus. Hence, *CitF3H* can serve as a potential candidate for genetic modifications for synthesizing beneficial flavonoids [151]. TFs related to citric acid, quinic acid and malic acid synthesis were identified [91]. TFs such as *CBF/DREB*, *MYB*, *AP2*, Zinc finger, *NAC* and *WRKY* in red fleshed orange [67] and *bHLH*, *MYB*, *ERF*, *NAC* and *WRKY* in pummelo [68] regulated carotenoid biosynthesis in citrus fruits. Additionally, *CsMADS6* was found to be directly regulating the lycopene b-cyclase and other carotenogenic genes in citrus [152]. The fruit cuticle is a determining factor in fruit quality, by preventing water loss from peel, disease tolerance and shelf life. ABA deficiency was found to influence the cuticle permeability during ripening [153], whereas MYB transcription factor GL1-like was involved in regulating wax synthesis in citrus [154]. Avocados are consumed for their high nutritious value. Expression levels of genes involved in carotenoid biosynthesis in avocado were found higher in pulp than compared to seed at different fruit developmental stages [155].

### 3.6. Bearing Characters

Alternate bearing, or biennial bearing, is a phenomenon of heavy bearing in one season followed by a low bearing, observed in apple, mango, citrus, avocado, litchi, olive, pistachio, etc. [156,157]. It is a major limiting factor in fruit production. Comparative gene expression and transcriptomic studies between ON and OFF crops enabled understanding of the flowering process in fruit trees. Among the reasons for the biennial phenomenon was the repressive effect of fruits on the flower initiation in apple. Genes such as Apetala 1 (*AP1*), Leafy (*LFY*), Fruitfull (*FUL*), Flowering Locus T (*FT*) and Squamosa Promoter Binding Protein-Like (*SPL*) and differentially methylated regions (DMRs) were found to affect the bearing in apple [158,159]. In citrus, fruit load had a negative impact on the expression pattern of flowering related gene homologue of FT, Suppressor of Overexpression of Constans 1 (*SOC1*), *AP1* and *LFY* in citrus [160]. Epigenetic regulation of flowering through the *CcMADS19* floral repressor was observed in citrus, which prevented the activation of the floral promoter *CiFT2* [69], whereas transcription factor homeodomain leucine zipper I (HD-ZIP I) was found to be repressing the activation of Flowering Locus C (FLC), which plays a major role in flower induction in citrus [70]. In mango, differential expression analysis revealed the involvement of *MiFT* and *MiAP1* genes and other positive regulators in regular bearing cultivar ’Amrapali‘ compared to alternate bearing cultivar ’Dashehari‘ [161]. Similarly, transcriptome analysis of regular (‘Neelam‘) and irregular (‘Dashehari‘) mango cultivars identified twenty-six alternate bearing genes, among which *SPL*-like gene, Rumani GA-20-oxidase-like gene and *LOC103420644* were significantly differentially expressed [162]. A recent computational study based on Arabidopsis and apple sequences identified twenty-nine putative genes of mango related to alternate bearing, which can further be used for studying the phenomenon [163].

Transcriptomic studies integrated with metabolomic and proteomic profiling have demonstrated the dynamic interplay of signaling and metabolic pathways governing fruit ripening at different stages of maturity in pulp and peel. Transcriptome and metabolome datasets can be used for network-based approaches of data mining and modelling to understand complex molecular processes in tropical fruits, as in citrus [164]. The metabolic pathway networks and correlation networks are useful to explore the relationships of the functional genes/metabolites. Recently, single cell omics has become a trend in biology and provides immense opportunity in studying fruit development and ripening mechanism in tropical fruits.

## 4. Shelf Life

Most of the tropical fruits are climacteric in nature, with a shelf life of only two to five days after harvest. Post-harvest treatments are employed to delay the ripening process and retain the fruit quality. In mango, hot water brushing of fruits upregulates genes involved in biotic resistance, colour development and lenticel discoloration [165]. Similarly, hot water treatment (HWT) increased the expression of genes involved in metabolic processes of sucrose, starch and cell wall polysaccharide [166], whereas mild high hydrostatic pressure (HHP) upregulated the carotene synthesis pathway genes [167].

Many genomic, metabolomic and proteomic studies have demonstrated a number of changes in primary and secondary metabolism in fruits in response to exogenous ethylene and ethylene inhibitors [168]. Exposure of fruits to ethylene enhances the ripening process of softening, aroma production and colour change to yellow. In fruits, 1-Methylcyclopropene (1-MCP) treatment inhibited the ethylene production, respiration rate, weight loss, soluble solid/acid ratios, lycopene, anthocyanin and carotenoid synthesis [169]. Additionally, 1-MCP treatment reduced the production of volatile esters and delayed sugar accumulation in banana. Furthermore, improper use of 1-MCP treatment resulted in stay-green disorder in banana, where the peel stayed green while the pulp ripened. The transcriptomic landscape developed in bananas with a ripening disorder revealed the inconsistency in expression of several ripening related genes [170]. In citrus, 2,4-dichlorophenoxyacetic acid (2,4-D) is widely used to maintain the post-harvest fruit quality and extend the shelf life. Transcriptomic and metabolomic profiling of peel after treatment with 2,4-D revealed the involvement of TFs such as *AP2/ERF*, *WRKY* and *NAC*s and increased hormone and defense related genes [171].

In papaya, long term 1-MCP treatments sometimes result in fruit ripening disorder by rendering a rubbery texture to the pulp. The transcriptome of long term 1-MCP treated rubbery pulped fruits was enriched with cell wall metabolism and hormone signal pathways along with lignin accumulation, suggesting their role in ripening disorder [172]. In addition, long term 1-MCP treatment inhibited the interaction and expression of *CpEBF1* and *CpMADS1/3* genes, resulting in non-activation of cell wall degradation-related genes, thereby leading to fruit softening disorders [173]. Furthermore, non-coding miRNAs such as *cpa-miR390a* targeting ethylene and *cpa-miR396* targeting auxin signalling pathways were found to be regulating the ripening process in 1-MCP treated fruits [102]. A comparative metabolome and transcriptome analysis further revealed that prolonged 1-MCP treatment drastically altered metabolites such as flavonoids, lipids, phenolic acids, alkaloids and organic acids in papaya fruits [9]. Metabolome cum proteome profiling of ethylene treated papaya fruits for 6 h was shown to have increased levels of GABA and volatile compounds such as terpene linalool and hastened the ripening process [174].

Sequencing of Thailand guava and Allahabad Safeda revealed SNPs in genes *ACO5*, *EIL3*, *EIN2*, *PL* and *ERF6* involved in ethylene biosynthesis and upregulation of transcription factors such as *TDR4/FUL1*, *MYB*, *MADS-RIN*, *ERF6* and *TAG1*, responsible for delayed fruit ripening and high shelf life in Thailand guava [73]. Such SNP-based molecular markers designed for fruit shelf life can complement the marker assisted breeding efforts in guava. In addition, downregulation of ACC synthase gene *pgACS1*, involved in the ethylene biosynthesis pathway, contributed to delayed ripening in harvested guava, unlike the normal ripening cycle [175]. This gene can be targeted for genetic modifications to create a guava cultivar with good keeping quality. During storage of dragon fruits, superoxide scavengers such as trypsin have been shown to delay fruit ripening by influencing genes involved in the flavanoid pathway [176], antioxidant signal pathways [177,178], redox enzymes [179] and stoma closure [180], with catechin gallate being the significant metabolite regulated by the treatment. After harvest, durian fruits undergo softening and dehiscence occurs in the peel. Expansins are hypothesized to be involved in fruit softening and, thereby, induce dehiscence. Studies have shown the upregulation of the expansin gene (*DzEXP1*) and ethylene receptor genes (*DzETR1* and *DzETR2*) during ethylene application and vice versa during 1-MCP treatment in durian fruit pulp [181,182]. The information generated from the above studies would help in formulating chemical treatments and storage conditions to extend the keeping quality of fruits.

## 5. Abiotic Stress

Environmental stress conditions such as drought, salinity, heat and flooding can cause severe disturbances in development and yield parameters, affecting the quality of fruits in tropical trees. Different omics approaches have been utilized to study the responses of crops to abiotic stress [183]. High temperatures were found to induce internal tissue breakdown in mango fruits [184]. Fruits had shown increased expression of abiotic and senescence related genes in response to heat stress at 44 °C [185] and also during chilling stress [186]. Transcriptome analysis facilitated the identification of 18 *eIF* genes (eukaryotic elongation factor) that were expressed under salinity, osmotic and low temperature (2 °C) stress [49]. Computational analysis using EST and RNA sequence databases in mango established non-coding RNAs such as miRNAs and lncRNAs to be involved in growth and development, ripening, transcription regulation and stress responses [96,99] (Table 2). Furthermore, *bHLH* TF genes and WD40 protein family members regulating abiotic stress responses in mango, citrus and papaya can provide the knowledge for understanding the responses during salt, drought, alkaline and cold stress conditions [50,51,71].

Chilling injury on fruits, other than causing physical damage, can also dramatically reduce fruit flavour. Storage of banana fruits at 5 °C and 20 °C caused chilling injury by negatively affecting the fruit appearance, and also reduced production of volatiles [187]. Further, comparative transcriptome and proteome analysis of low temperature-treated banana facilitated identification of MYB TFs [44] and cold stress responsive 12,462 lncRNAs [101]. Similar types of studies helped to decipher the molecular mechanisms in citrus in response to low temperature for maintaining fruit quality and storage life [188]. Low temperature (10 °C) increased the expression of carotenoid of biosynthetic genes (*CitPSY* and *CitVDE*) and decreased the catabolic gene expression (*CitNCED2* and *CitNCED3*), thereby increasing the total carotenoid content in citrus juice sacs [189]. Citrus fruits are highly sensitive to salt stress, and studies identified genes related to cell wall loosening and stiffening linked in salt stress [72], whereas miRNAs and 28 PHAS genes were found linked to alkaline stress [71].

Screening of drought tolerant and susceptible landraces of guava using SRAP and ISSR markers have shown to be effective and promising in analyzing germplasms tolerant to drought [190]. On the other hand, 40 potential microRNAs related to salinity stress and their target transcripts were characterized in guava [103]. Transcription factors regulating drought stress tolerance were examined in papaya and belong to TF families such as *CpHSF*, *CpMYB*, *CpNAC*, *CpNFY-A*, *CpERF* and *CpWRKY* [63,191]. Though there is no genetic information on drought stress related mechanisms in jackfruit, SSR markers developed and QTLs identified for drought tolerant root traits in mulberry can be studied for assessing the jackfruit germplasm, as they showed 79.25% transferability to cross species [192]. In dragon fruits, abscisic acid receptor PYL8, amino acid permease 8 and C2H2 zinc finger proteins were reported to be upregulated during drought stress conditions [193]. The catalase gene of dragon fruit (*HuCAT3*) and proteins involved in chloroplast and mitochondria metabolism were documented to play a crucial role in abiotic stresses such as cold, drought and salt stresses [194,195]. In addition, the ethylene response factor gene (*HuERF1*) of white pulped, salt tolerant *Hylocereus undatus* enhanced the antioxidant capabilities, thereby conferring salt tolerance in dragon fruit [78].

## 6. Biotic Stress

Ripening brings about changes in the fruit firmness, which makes it susceptible to pests and infections from pathogens during the final stages of ripening or during storage. In guava, microsatellite markers were employed to identify tolerant and susceptible accessions for the bark-eating caterpillar (*Indarbela tetraonis* Moore) of guava, which can be used as potential parents in genomic assisted breeding against the insect attack [196]. Similarly, SSRs were used for screening of segregating mapping populations and selection of hybrids with better resistance towards southern root-knot nematode [197]. Stem canker caused by *Phytophthora palmivora* is an important disease affecting the durian plantations. The development of SCAR Markers specific to Phytophthora resistance in durian [198], identification of SSR loci associated with durian die-back resistance [199] and Mildew Locus O (MLO) associated with powdery mildew disease [200] can be employed in breeding for disease resistance in Durian species.

The major tropical fruit crops—mango, papaya, banana and minor fruits such as avocado, guava and dragon fruit—are susceptible to anthracnose infection caused by *Colletotrichum* sp., both at pre-harvest and post-harvest stages. This fungus, apart from infecting several parts of the plant, also infects fruit during post-harvest or during the ripening stages, posing major economic losses. Multiple markers have been utilized to characterise different species of *Colletotrichum* causing anthracnose [201]. Symptoms typically include the formation of black to dark brown sunken lesions on the fruit surface, reducing fruit quality and marketability. Transcriptome analysis of *Colletotrichum gloeosporioides* infected mangoes revealed the active role of 35 defense related genes related to the gene families *EFR*s, *NBS-LRRs*, *NPR*s, etc. [202]. In the case of banana, molecular approaches were followed to understand the host resistance against *Colletotrichum musae* and identified TFs *MaNAC5*, *MaWRKY1* and *MaWRKY2* as transcriptional activators regulating *MaPR1-1*, *MaPR2*, *MaPR10c* and *MaCHIL1* genes involved in salicylic acid and MeJA induced pathogen resistance [47] (Table 1). Similar transcriptomic analysis was reported in avocado in response to the *Colletotrichum* pathogen [203,204]. Further genome-wide analysis of basic helix–loop–helix (bHLH) TFs [205] and comprehensive transcriptomic analysis of sugar transporter (*SWEETs—*Sugars Will Eventually be Exported Transporter) [206] studied the candidate genes related to fruit ripening and biotic stress in banana.

*Penicillium digitatum* affects post-harvest storage in citrus fruit and its infection induces the G-protein and RLK signal pathways and enhances the transcription of peroxidase and NBS–LRR. Comparative metabolomics and transcriptomic profiling revealed that the defense responses were activated by *WRKY*, *MYB* and *AP2/ERF* via jasmonic acid and ethylene pathways [207], while a BZIP TF of sweet orange *CsBZIP40* was reported for citrus canker resistance by activating *PR* genes through SA signalling pathway in the presence of *NPR1* [208]. Similarly, RNA sequencing of citrus fruit in response to the infection of three fungal pathogens viz. *P. digitatum*, *P. italicum* and *G. citriaurantii* revealed the differential expression of genes related to host innate immunity, secondary metabolite synthesis and hormone signalling [209]. Similarly, studies on dragon fruit infected with canker disease revealed enriched *LRR*, *MYB*, *NAC* and *WRKY* family genes [210,211].

Papaya sticky disease (PSD) is a combined infection of papaya meleira virus (PMeV) and papaya meleira virus 2 (PMeV2). Transcriptomics of PSD infection at pre-flowering accumulated negative regulators of SA signalling, consisting of NPR1-inhibitor (*NPR1-I/NIM1-I*), genes coding for UDP-glucosyltransferases (UGTs) and ethylene pathway, whereas post-flowering infection caused the downregulation of *PR-1* gene and the induction of BSMT1 and JA metabolism related genes [212]. Transcriptome profiling of PRSV resistant transgenic papaya identified TFs such as *MYB*, *WRKY*, *ERF*, *NAC* and nitrate and zinc transporters involved in ABA, SA and ethylene signalling pathways [213]. Extensive study of the molecular level interaction of host–pathogen, signalling responses and metabolites expressed during biotic and abiotic stress conditions can help in the development of markers for screening germplasm and progenies, genetic modifications and breeding of new cultivars with enhanced tolerance to various stresses.

## 7. Genomic Assisted Breeding Strategies in Tropical Fruit Crops

Breeding in fruit crops with improved fruit characters requires a thorough understanding of the genetic architecture of the fruit quality traits, requiring population segregation, a wide and diverse germplasm collection, diversity analysis and genetic studies by vast and exhaustive phenotyping over many years.

### 7.1. Diversity Analysis

Diversity studies will allow a wider understanding on the genome accessions with variable fruit characters and their origins. As the sequencing technologies improved, the generation of genomic knowledge of fruit crops progressed from application of molecular markers for genetic diversity analysis [214,215] and DNA fingerprinting [216] to the generation of high throughput sequencing based SNPs, TRAPs (Target Region Amplification Polymorphism) and SSR markers [217,218]. SNP genotyping [219] was developed based on transcriptome and whole genome sequence information in mango and banana. Considerable efforts had been made to identify potential markers linked to fruit characters. Among the available molecular markers, the SSR markers are more efficient due to their multiallelic nature. Microsatellite markers have wider applicability in terms of their transferability to other species and genera in tropical fruit trees. Such intergeneric and interspecific markers aid in diversity analysis and help generate genomic SSRs in the target crops with limited genetic information [220,221]. Recently RADseq, DARTseq and GBS (Based on NGS technology) provide high genome coverage and accuracy for genetic analysis [222].

In the case of banana, the world’s largest germplasm collection was maintained at the Bioversity International Transit Centre (ITC), Belgium [223]. Genetic diversity of carotenoid-rich bananas using Diversity Arrays Technology (DArT) assisted in selection of carotenoid rich bananas [224]. Functionally relevant SSR markers (FRSMs) were developed for germplasm characterization, genetic diversity studies and comparative mapping in Musa spp. and other monocot species [222]. Evaluation of 100 Indian musa accessions for nine mineral elements in their fresh fruit pulp (FFP) demonstrated genetic variability of 4.7-fold for K (Potassium) and Mg (Magnesium) and 111.1-fold for Ca (Calcium). From the study, 20 commercial cultivars were placed in the top 10 based on their mineral contents [225]. In papaya, an atlas/map of SSR markers was developed and validated by using gene predictions and functional annotation data. The atlas consists of 160,318 SSRs, of which 21,231 were from genic regions (inside exons, exon-intron junctions or introns). They have listed 300 genes (comprising 785 SSRs) involved in fruit ripening, validated 73 SSR markers (including 25 fruit ripening genes) with 100% amplification rate and showed 26% polymorphism between the parental genotypes [226].

Molecular breeding strategies in guava are relatively limited compared to other major tropical fruits. Nonetheless, molecular markers were utilized for characterization [227,228] and varietal identification [229]. Using AFLP markers, diversity analysis studies grouped the white fleshed and pink fleshed guava varieties in separate groups [230]. Recently, a major improvement in understanding the molecular mechanisms and genes involved in controlling the ascorbic acid accumulation and fruit softening was brought about by the whole genome sequencing of *Psidium guajava* [231]. In the case of jackfruit, genetic diversity analysis has shown that white pulp and firm textured fruit varieties were more genetically diverse compared to red and yellow pulps and soft textured fruits, respectively [232]. A notable addition to the genetic information of jackfruit is its draft genome assembly. The assembled genome contained 51% of repeat sequences and 35,858 protein coding genes. The gene families in starch synthesis were found to be more expanded in *Artocarpus heterophyllus* than its related species [233] (Table 4).

In dragon fruit, grouping of accessions with pink and white pulp colours has separately been achieved through ISSR [247,248] and SSR markers [249]. SSR markers were successfully used to characterise durian cultivars based on blossom end area and fruit spine density [250]. Chromosome level genome assembly in durian points out the expansion of genes involved in production of volatile sulphur compounds (VSCs) contributing to their unique flavour [246]. Chloroplast genome assembly and plastome sequences of durian are also made available, which can complement the genetic information necessary for breeding [251,252]. A single major gene, *CpCYC-b*, responsible for papaya fruit flesh color, was identified and a SCAR marker (CPFC1) was developed [253], facilitating identification of progenies based on pulp colour.

Interspecific crosses have been employed over the years for crop improvement, to generate progenies with improved fruit traits and stress tolerance in tropical fruit trees. However, incompatibility among the species is a major concern in fruit crop breeding, such as in banana, guava and papaya. Interspecific crosses between wilt and nematode resistant, polyploid *P. cattleyanum* and diploid commercial cultivars of *Psidium guajava* are faced with problems of incompatibility and low efficiency of methods in selecting the segregating mapping population introgressed with resistance genes [254,255]. SSR markers developed specifically for *P. cattleyanum* [256] and SNP markers with a potential to identify the hybrids generated through interspecific crosses in *Psidium* sp. [257] can help ease the breeding problems. Such species-specific markers for tropical fruits can facilitate the evaluation of hybrids at the seedling level for their genetic variability and required fruit quality traits.

Assessing genetic diversity is a first step towards developing an effective genetic improvement program. A successful fruit crop improvement program requires morphological and molecular characterization and preservation of diverse accessions with respect to the economically important traits or fruit related traits. Advancement in genomic approaches such as whole genome sequencing and annotation, transcriptomics, proteomics and metabolomics and bringing such data to global platforms facilitates their access by entire scientific community. Using such data platforms, it is possible to capture the polymorphism at different levels and to complement the genetic diversity studies with respect to fruit traits.

### 7.2. QTLS/Genes Related to Fruit Traits

Identification of markers, QTLs or genes related to the trait of interest is a prerequisite for genome assisted breeding (GAB) facilitated through multi omics techniques. From molecular characterisation and DNA fingerprinting, molecular markers are now being efficiently utilized for population structure analysis, fruit trait-based clustering and gene tagging. Marker assisted selection (MAS), marker assisted introgression (MAI) and marker assisted backcrossing (MAB) strategies have been developed to reduce the time and labour required for mapping population generation, phenotyping and cultivar identification. Quantitative trait loci (QTLs) associated with guava fruit traits such as fruit length, fruit width, fruit weight, seed weight, seed strength, seed number, inner pulp thickness, vitamin C, plant height and yield were located among the 11 linkage groups representing the haploid genome number of guava [258,259]. Linkage maps were developed using different F1 segregating mapping population in mango utilizing AFLP, SRAP, ISSR, SNPs and SLAFs [8,260,261].

In papaya, the genotyping-by-sequencing (GBS) approach was used to detect QTL for fruit quality traits, 219 SNPs were used to construct linkage map across 10 LGs and covering 509 centiMorgan (cM) and a total of 21 QTLs were identified for seven key fruit quality traits (flesh sweetness, fruit weight, fruit length, fruit width, skin freckle, flesh thickness and fruit firmness) [262]. In papaya, using whole-genome genotyping (WGG), it was reported that 106 ripening-related genes were associated with 460 SNP/Indel variants, and they can be converted as molecular markers to aid genetic mapping and diversity studies and also to facilitate MAS for specific fruit traits [263]. QTL analysis showed four QTLs associated with fruit weight, one QTL associated with sugar content and three QTLs associated with peel puffing in mandarin [264]. DArTseq markers were also used for QTL mapping of citrus fruit quality [265].

Polyembrony in mango was found to be associated to linkage group 8, along with a possible linkage of SNP markers to vegetative traits such as bloom and blush characters and fruit traits such as beak shape and pulp colour [8]. Whole genome assembly of mango using different high throughput sequencing techniques has been attempted by various research groups [97,234,235,236]. Integration of RNA sequence information with the genome assembly paved the way for identification of non-coding RNAs, two QTLs for fruit weight and chalcone synthase genes involved in flavanoid biosynthesis. A whole genome assembly of dragon fruit validated the earlier studies and further facilitated the construction of high-density linkage map, using 56,380 SNP markers employing GBS, and identified 43 genes and 80 transcription factor families involved in betalain synthesis [74] (Table 4). Allelic variation in carotenoid metabolic genes was characterized by eQTL analysis and found that there were seven PSY alleles, seven HYb alleles, eleven ZEP alleles, five NCED alleles and four alleles for the eQTL that control the transcription levels of *PDS* and *ZDS* among the ancestral species [266]. The discovery of genome-wide SNPs for characterizing the diversity of the avocado germplasm is a powerful and cost-effective approach for a breeding program [267]. The largest avocado mapping populations and an extensive germplasm collection are located at the USDA–ARS Subtropical Horticulture Research Station (SHRS) in Miami, Florida. The development of the first set of avocado genetic markers was achieved based on SNP variation in expressed genes [268]. They have utilized RNA sequencing to build a reference transcriptome and identified SNPs. These genomic resources can be efficiently utilized to assess the avocado germplasm genetic diversity worldwide.

### 7.3. Application of GWAS/GS in Marker Assisted Breeding

Next generation sequencing based genotyping-by-sequencing (GBS) enabled high throughput genotyping through the identification of enormous amounts of single nucleotide polymorphisms (SNPs), which in turn facilitated the genome wide association (GWAS) and genomic selection (GS) in heterozygous temperate fruit crops such as apple, pear [269], prunus [270] and plum [271], and tropical fruit crops such as citrus and banana. Multivariate models have been developed to improve the predictive efficiency of GS models in fruit crops [272].

The exploitation of genome-assisted approaches in tropical crops would quicken the breeding efforts for fruit quality traits, as genomic selection enables the prediction of the phenotype at the seedling stage and thereby reduces the long wait for fruit phenotyping at maturity and the generation time in plant breeding programmes [273]. GWAS have been applied in identifying the QTLs for flowering, aroma volatiles, sugar, ripening period, fruit texture and harvest date in apple [274,275] through genome wide molecular markers and metabolite data. In peach, marker assisted mapping of QTLs for almond fruit type, flat fruit shape, juiciness, blood flesh, titrable acidity, soluble solid content, etc. have potential to assist MAS and MAI strategies [276,277]. This was further complemented using genome assisted prediction models for selection of fruit weight, sugar content and acidity [278]. Fine mapping of candidate genes in fruit species can be utilized for developing superior varieties. In the case of peach, fine mapping of powdery mildew resistance gene Vr3 facilitated MAI of the same into elite peach cultivars [279], whereas fruit skin trichome trait locus mapping promoted the MAS in progenies [280]. Further, integrated pedigree based analysis and GWAS techniques reviewed earlier [281] give us a glimpse of the potential of these strategies in increasing the breeding efficiency in fruit crops. MAS was applied successfully to incorporate CTV (Citrus Tristeza Virus) resistance of trifoliate orange into transgenic citrus, over expressing early flowering genes [282] and blue mold resistance locus into transgenic apple line T1190, expressing early-flowering gene, thereby reducing the breeding cycles [283]. Similarly, QTLs for seventeen citrus fruit quality traits such as peel colour, fruit weight and firmness, identified using association mapping and regression models [284] and 206 QTLs associated with volatile production [285], can be applied in breeding methods such as MAS, MAB and MAI in citrus. In grapes, a molecular marker for seedlessness has been applied for MAS in embryo rescued hybrids [286].

In banana, GWAS was utilized to identify genes responsible for seedless phenotype and found 13 candidate genomic regions potentially associated with the seedless phenotype (i.e., parthenocarpy combined with female sterility) [287], and also identified 28 genes related to flavonoid biosynthesis and its regulation in the peel and pulp [288]. Fruit filling in banana was found to be associated with multiple major QTLs and bunch weight, and its related traits were associated with chromosome 3 [289]. Further, the BayesB model helped predict fruit traits such as fruit filling and fruit bunch with high predictive values [290]. Such genomic information on fruit traits in bananas can support and quicken the breeding process. Similarly, QTLs related to fruit traits were identified using SNPs in tropical trees such as mango [8], avocado [268] and papaya [262] which can expedite the breeding efforts. Previous studies had emphasised the cost-effectiveness of MAS in peach and apple breeding programs compared to traditional breeding approaches [291,292]. Hence, the combined power of marker assisted GWAS and model-based GS can be adopted to reduce time and cost in crop improvement.

New innovations in sequencing technology and improvement in computational methods have revolutionized genomic information generation, and the most recent is the creation of the pan-genomes databases, which is valuable for crop improvement. To date, SNPs were utilized for GWAS and QTL identification using a single reference genome. However, single molecule, long read sequencing and resequencing allows the identification of structural variations (SVs) within the species of a crop and characterization of the pan-genome, pan-transcriptome and super pan-genomes representing entire sequence diversity, thereby mining the genetic determinants of a phenotype and advancing the genomic prediction [293,294]. Transposable element mediated ncRNA discovery in crops can also become more efficient using pan-genomes, further aiding the studies on regulation of plant metabolism [295]. Pan-genomes of fruit crops such as apple and banana have laid the foundation for future studies which can accelerate the functional genomics and crop improvement in fruit trees [296,297].

## 8. Genetic Engineering in Fruit Crops

Genetic engineering (GE) is meant to tweak only the intended changes in the cultivar, keeping the other traits unchanged. The approach is a complementary option used for incorporating useful traits to bypass the long generation time, polyploidy and sterility of most of the cultivated varieties, and can also solve post-harvest crop loss and improve food security, as well as can be applied for biofortification of nutraceuticals. Genetic engineering approaches such as transgenics, cisgenics, intragenics, RNAi technology and, recently, genome editing tools such as CRISPR-cas9 were reported to modify the fruit quality traits.

### 8.1. Transgenics

Genetic engineering in banana crops is advantageous because most of the banana cultivars are essentially sterile; transgene flow and the outcrossing of modified genes into wild Musa species are highly unlikely and virtually impossible in other triploid cultivars [298]. Few successful transgenics in fruit crops (Table 5) include the generation of GE banana with biofortified pro-vitamin A (phytoene synthase enzyme (PSY) and iron content (Ferritin gene from soybean) [299,300]. Grand Naine banana was transformed using a triple gene construct, where endochitinase gene *ThEn-42* was taken from *Trichoderma harzianum* and grape stilbene synthase (*StSy*), and superoxide dismutase Cu, *Zn-SOD* was taken from tomato [301]. It was reported that the transgenic was tolerant to sigatoka and gray mold/ blossom blight (*Botrytis cinerea)* [302]. Papaya chromoplast-specific lycopene β-cyclase, (*CpCYC-b*) controls fruit flesh color where two alleles, yellow fleshed allele (dominant) and red fleshed allele (recessive), were fully sequenced [253]. A two bp insertion was identified within the red fleshed coding region of *CpCYC-b*, producing a frameshift mutation resulting in a premature stop codon and a truncated coding region in the red-fleshed *CpCYC-b* allele. A papaya plant infected with papaya ring spot virus (PSRV) shows ringspot on its fruit, which reduces fruit quality and also affects the fruit set [303]. Transgenic virus-resistant papaya ‘Rainbow’ was developed in Hawaii and was rapidly adopted by farmers. Jia et al., (2017) [304] have used RNAi technology to develop coat protein (CP) mediated PRSV-resistant transgenic papaya for the USA and Taiwan. Other genetic characteristics of fruit quality traits, tree physiology and abiotic stress tolerance were altered through this transgenic approach for apple, banana, papaya and pineapple [305,306].

Fruit ripening can be delayed with delayed climacteric respiration and reduced synthesis of ethylene. In banana, two MADS-box (ripening genes-*MaMADS1* and *MaMADS2*) were known to regulate fruit ripening and used to improve fruit shelf life. Transgenic bananas developed through antisense or RNAi technology, *MaMADS* altered plants exhibited delayed ripening and extended shelf-life. Further, these transgenic banana fruits responded to exogenous application of ethylene [310]. Similarly, red banana (*Musa acuminata* L.) was transformed with sense and anti-sense constructs of the *MaMADS36* transcription factor gene (*MuMADS1*, *Ma05_g18560.1*). *MaMADS36* directly binds to the promoter of *MaBAM9b* regulating enzyme activity and starch degradation during ripening, thus delaying fruit ripening and extending the shelf life. The transgenic papaya with delayed ripening was developed through the antisense technology for 1-aminocyclopropane-1- carboxylic acid (ACC) synthase (ACS) (suppressing the production of ethylene during ripening by blocking the activity of ACS). When transgenic papaya was compared with control papaya, the composition was not altered with respect to some fruit characters such as β carotene, vitamin C and other proximate fruit composition [316]. Though there is a very limited research related to transgenics in fruit crops, the development of ‘Arctic’, a non-browning apple [307] and Pinkglow™, a pink fleshed pineapple [308] with improved fruit quality traits can serve as proof of concept studies that can be adapted in tropical fruit trees.

Intragenics and cisgenics were developed as alternative tools to the transgenic approach. Cavendish banana was modified through a cisgenic approach for enhancing the level of provitamin A and to increase the resistance to Panama disease [298]. Potatoes resistant for late blight and black spot bruising, developed by using Innate^®^ technology, are the only intragenic crop species that has been approved for commercialization [317]. In citrus fruits, bitterness and an extremely sour and acidic nature significantly impact the sensory quality of citrus products. Li et al. (2019) [318] have reviewed the major genes involved in pathways influencing the sweet, bitter or sour taste and discussed the possible approaches to modify citrus taste through genetic engineering. Many genes/TFs have been characterized for fruit quality traits and can be targeted by employing genetic engineering approaches to improve the tropical fruit crops.

### 8.2. Genome Editing/Gene Editing/CRISPR/Cas9

Genome editing is an efficient tool to introduce precise mutations in plants and also a better alternative to conventional breeding methods without the extensive backcrossing and selection. Presumably, regulations are going to be simple for genome edited crops compared to GMOs because of the lack of transgene in the edited crops. Genome editing platforms are applicable to study gene function and mechanism of regulation by the generation of mutant crops and serve as excellent tools for crop improvement. Using genome editing tools, it is possible to remove undesirable chromosomal DNA, upregulation or downregulation of desirable genes and the insertion of novel coding sequences. The CRISPR/Cas9 genome editing system is becoming highly effective in several dicotyledonous and monocotyledonous species [319]. This technique has great potential for improving fruit qualities in tropical fruit crops.

To examine the effectiveness of gene editing in Cavendish banana, which accounts for about 47% of global production, Naim et al. (2018) [320] have targeted the phytoene desaturase (*PDS*) gene using the CRISPR/Cas9 system. Similarly, the CRISPR/Cas9 system was used to target the PDS gene in sweet orange leaves with a mutation efficiency of 3.2 to 3.9% with no quantifiable off-targets [314,321]. The *PDS* gene has been used in a variety of plant species as a convenient indicator for CRISPR/Cas9-mediated gene knockouts [322,323]. In citrus, editing efficiency was improved by using CRISPR-LbCas12a editing of *PDS* [324], whereas Duncan grapefruit canker susceptibility gene *CsLOB1* was modified with a mutation frequency of 23.80 to 89.36% with no off-targets [314]. Employing the glycine tRNA processing system for delivering a polycistronic gRNA generated a 100% PDS modification success rate, primarily in the form of small nucleotide indels upstream of the PAM consensus sequence [320]. This CRISPR/Cas9 editing system can be used for improving fruit quality traits. CRISPR/Cas9-mediated genome editing was used to promote the shelf life of banana fruit by editing *MaACO1* (amino-cyclopropane-1-carboxylate oxidase 1). The mutant fruits developed by editing exhibited reduced ethylene synthesis and extended shelf life under natural ripening conditions [311]. The β-carotene enriched fruit was developed using CRISPR/Cas9 in Cavendish banana after targeting the lycopene epsilon-cyclase (*LCY* *ε*) gene [312].

Viral diseases that affect a banana crop include the banana bunchy top virus and banana streak viruses, which are widespread and economically damaging. The CRISPR system evolved to detect targeted gene sequences with Cas9, Cas12, Cas13 and Cas14 enzymes, thereby, CRISPR/Cas-based genome editing can be explored for virus diagnosis and developing resistance against banana viruses [325]. In papaya, CRISPR/Cas9-mediated mutations in S-genes conferred resistance to PRSV [313]. In contrast, the mutation of *PpalEPIC8* (Cysteine protease inhibitor) in *P. Palmivora*, a fungal pathogen, was created to study the papain mediated resistance in papaya [326]. Similarly, CRISPR/Cas editing can be applied for the fruit quality parameters in tropical fruit trees, thereby reducing the breeding cycle and improving fruit quality. Advances in molecular breeding approaches, particularly genome editing technologies, provide great opportunities to develop new fruit cultivars more rapidly. However, there are constraints, benefits and commercial opportunities that exist in transgenic and genome-edited fruits, and these were reviewed elaborately by Gomez et al. (2021) [10]. Genome editing techniques can also be employed for generating novel traits in fruit crops. Recently, in wild tomato, the *S. pimpinellifollium* genome was edited to improve fruit number, size, shape, nutrient content and plant architecture [327]. Such a possibility does exist for many tropical fruit species using genome editing tools.

## 9. Databases

The development of crop specific databases based on transcriptome and whole genome data will help make the genomic information of these fruit crops easily accessible to breeders. A transcriptome based SSR database, MusatransSSRDB, facilitates the selection of SSR primers for a specific objective and also provides information on in silico polymorphic 2830 SSRs. In silico primer analysis helps to avoid the selection of monomorphic primers [328]. Approximately 1500 proteins in citrus fruit juice sac cells were quantified at three developmental stages. As a result, a comprehensive sequence database of citrus genes, ESTs and proteins, named iCitrus, has been established for proteomic research [329]. Similar genomic databases are available for mango, banana, guava, papaya and durian (Table 6).

## 10. Conclusions

Due to recent advances in sequencing techniques, knowledge on genes governing the fruit development, ripening mechanism and aroma development in the tropical fruit crops has vastly expanded. However, a systematic approach to developing sequence information on the intricate pathways of fruit ripening, shelf life and stress responses in tropical fruits is still less frequently implemented compared to temperate fruits. An integrated omics approach can provide the functionally validated genomic information necessary for breeding programs. Genome wide association studies utilizing the multi-omics data would bridge the gap for identification and association of the multigenic traits that govern fruit characters and stress tolerance in tropical fruits. Further, the genomic selection approaches eradicate the necessity of developing a mapping population, thereby reducing the time and space required for maintaining fruit orchards. Information generated in other model crops such as apple and tomato can be used for tropical fruit crops to improve fruit quality. A combined systems biology approach will benefit tropical fruit breeding by effectively reducing the generation cycle. Breakthrough technologies such as single cell omics and CRISPR mediated genome editing can be a boon for developing new cultivars within a short duration, at ease and with better accuracy in tropical fruits. There is an urgent need to develop tissue culture regeneration protocols for many tropical trees to exploit genetic editing technology.

## Figures and Tables

**Table 1:** Transcription factors involved in fruit ripening and biotic and abiotic stress conditions.

Crop	Gene Family	Number of Transcription Factors	Fruit Traits	Reference
Banana	basic leucine zipper (*bZIP*) TF—*MabZIP93*	1	Fruit ripening	[37]
	*MabZIP*	121	Fruit development, ripening and abiotic stress	[39]
	*MabZIP74*	1	Fruit ripening	[38]
	*MaMADS2-box*	1	Fruit ripening	[33]
	*MaDof*	4	Fruit ripening	[40]
	*MaDREB1-MaDREB4*	4	Fruit ripening	[41]
	*MabZIP4 & MabZIP5*	2	Fruit aroma	[42]
	serine/threonine protein kinases—*MAPKK* and *MAPKKK* genes	10 77	Fruit development, ripening and responses to abiotic stress	[16]
	Basic helix–loop–helix (*bHLH*)—*MaTCP5*, *MaTCP19* and *MaTCP20*	3	fruit softening	[34]
	*MaNF-Ys*	13	Fruit ripening	[43]
	*MYB* *MYB3* *R2R3- MYB*	9 1 285	Fruit softening	[36,44,45]
	*MaBZR1/2*	1	Fruit softening	[36]
	*MaSPL16*	1	Carotenoid biosynthesis	[46]
	*MaNAC5*, *MaWRKY1* and *MaWRKY2*	1	Biotic stress	[47]
Mango	*MADS*-*box* *EIN3* factors *APETALA2-like* TFs Ethylene response element factors	39 4 4 1	Fruit softening	[29]
	RabGTpase	-	Fruit softening	[48]
	Eukaryotic translation initiation factors (*eIF*s)	18	Abiotic stress	[49]
	*WRKY*	10	Mango malformation	[17]
	*MibHLH*	212	Hormone responses, abiotic stress	[50]
	*WD40 family*	315	Protein–protien interaction, abiotic stress response	[51]
Papaya	*CpAP2/ERF—RAP2.4*	1	Tolerance to cold and heat stress	[52]
	*CpGRF1–8*	8	Fruit ripening and abiotic stress	[53]
	*CpMADS4* and *CpNAC3*	1	Fruit ripening	[54]
	*CpMYB1*, *CpMYB2*	1	Fruit softening	[55]
	*CpARFs* *CpEIL1*	11 1	Fruit ripening	[56,57]
	*CpSPLs*	14	Fruit ripening	[58]
	*cpNAC1*, *cpMYB1*, *cpMYB2*, *CpbHLH1*, *CpbHLH2* and *CpEIN3a*	1	Carotenoid biosynthesis in fruits	[55,59,60,61]
	*CpbHLH*	73	Abiotic stress	[62]
	*CpHSF*, *CpMYB*, *CpNAC*, *CpNFY-A*, *CpERF* and *CpWRKY*	1	Drought stress	[63]
Citrus	*C2H2*, *Dof*, *bHLH*, *ERF1*, *MYB*, *NAC*, *LBD*, *ARF1* and *TGA9*	16	Late fruit ripening	[64]
	*ERF1*, *ARF1* and *TGA9*	1	Late fruit ripening	[65]
	*MADS*	1	Early fruit ripening	[66]
	*CBF/DREB*, *MYB*, *AP2*, *Zinc finger*, *NAC*, *WRKY*, *bHLH* and *ERF*	-	Carotenoid biosynthesis	[67,68]
	*CcMADS19* and homeodomain leucine zipper I (*HD-ZIP I*)	1	Alternate bearing	[69,70]
	*ERF* *bHLH* genes *C2H2* genes *WRKY46* *HRA1* and Chitin-inducible gibberellinresponsive protein1	6 2 2 1 1 1	Alkalinity stress	[71]
	*WRKY*, *NAC*, *MYB*, *AP2/ERF*, *bZIP*, *GATA*, *bHLH*, *ZFP*, *SPL*, *CBF* and *CAMTA*	-	Salinity stress	[72]
Guava	*WRKY*, *AP2*, *bHLH*, *PHOR1*, *MYB* and *C2C2.CO*	-	Fruit ripening	[21]
	*TDR4/FUL1*, *MYB*, *MADS-RIN*, *ERF6* and *TAG1*	-	Delayed fruit ripening	[73]
Dragon fruit	*MYB*, *bHLH* *WRKY*	185 168 80	-	[74]
	*HpWRKY44* and *HpWRKY3*	1	Betalain biosynthesis	[75]
	*HpNAC*, *HpGSTs* and *MYB*	1	Betalain biosynthesis	[76,77]
	*HuERF1*	1	Salt stress	[78]
Durian	DNA binding with one finger (*Dof*)	24	Fruit ripening	[79]
	Ethylene response factor (*ERF*) transcription factor	63	Fruit ripening	[80]
	Auxin response factors (*ARF*s)	15	Fruit ripening	[81]

**Table 3:** Genes and metabolites contributing to diversity of peel colour in tropical fruits.

Crop	Peel Colour		Genes Involved	Metabolites	Reference
Mango	Green	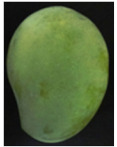	UDP-glucose:flavonoid-O-glycosyl-transferase (UFGT), dihydroflavonol 4reductase, anthocyanin synthase (ANS), chalcone synthase and basic helix loop helix (BHLHX)	All trans-violaxanthin butyrate	[123,124]
	Yellow	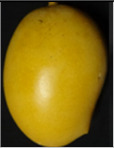	Carotenoid biosynthesis viz. lycopene-β-cyclase and violaxanthin-de-epoxidase	β-carotene and violaxanthin	[123]
	Red	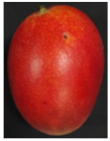	Anthocyanin biosynthesis genes viz. phenylalanine ammonia lyase (PAL) and p-coumarate 3-hydroxylase (C3H), flavanone 3-hydroxylase, anthocyanin synthase, MiC4H, Mi4CL2, anthocyanin synthase (MiANS) and UDP-glucose:flavonoid-O-glycosyl-transferase (MiUFGT2), flavonoid 3′hydroxylase and transcription factors MYB and basic helix loop	cyanidin-3-O-monoglucosides and peonidin-3-O-glucosides	[123,124,143]
Banana	Yellow	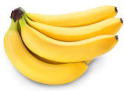	Carotenoid biosynthesis genes	Lutein, D-carotene, and E-carotene	[144]
	Red	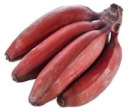	Anthocyanin biosynthesis genes	Rutinoside derivatives of cyanidin, peonidin, petunidin, and malvidin	[144]
	Purple Ex: M. itinerans	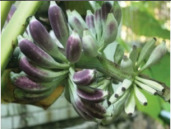	Anthocyanin biosynthesis genes and transcription factorssuch as MYB, bHLH, WD40 gene and R2R3-MYB	—	[128]
Papaya	Yellow (ArkaPrabhat)	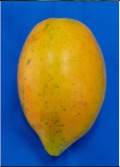	Carotenoid biosynthesis pathway genes such as PSY1, PDS1, ZDS, LCYB1, CHYB, LUT1, ZEP and VDE	lutein and β-carotene	[132]
Guava	Yellow (Allahabad Safeda)	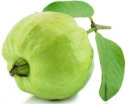	anthocyanin biosynthesis genes such as phytoene synthase and Aminocyclopropane-1-carboxylate oxidase 1-like	—	[21]
	Red (Apple colour)	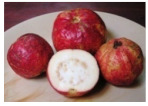	Phenypropanoid and lignin biosynthesis pathway genes and anthocyanin biosynthesis genes such as phenylalanine ammonia lyase (PAL), reticulin o-methyltransferase, glycerol-3-phosphate acyltransferase 5 (GPAT 5), peamaclein, CTP synthase-like, chloroplastic monodehydroascorbate (MDA), probable 2-oxoglutarate dependent dioxygenase AOP1 (2OG-AOP1) and methionine synthase (MS)	Reticulin o-methyltransferase	[21]
Citus	Yellow	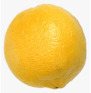	Carotenoid biosynthesis genes and TFCcGCC1 (Garp and coiled-coil)	β-carotene	[131]
	Red/dark orange	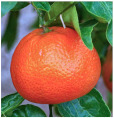	Carotenoid biosynthesis genes such as CsPSY, CsZDS, CsZ-ISO, CsBCH1 and carotenoid cleavage dioxygenases genes and TFs such as CsFUL2, CsTAGL1, CsRIN1, CsRIN2 and CsHY5	β –citraurinene, β -citraurin, phytoene and phytofluene	[129,145]
Dragon fruit	Green	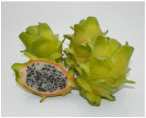	Carotenoid biosynthesis gene such as ZEP and Betalain biosynthesis pathway genes such as CYP76Ads	Gomphrenin-I, Cyanidin 3- O-malonylhexoside, Cyanidin chloride, Betanin, malvidin 3-o-galactoside and oenin chloride	[137]
	Yellow	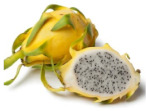	Betalain biosynthesis pathway genes such as TYDC and 2-aminoindan 2-phosphonic acid gene	Cyanidin 3- O-malonylhexoside, cyanidin O-syringic acid, 6-C-Hexosyl-hesperetin O-hexoside, citric acid, isochlorogenic acid A, verbascoside and luteolin-7,3′-Di-O-β-D-Glucoside	[137]
	Red	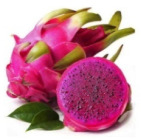	Betalain biosynthesis pathway genes such as CYP76ADs, Carotenoid biosynthesis gene such as phytoene synthases (PSY) and PLIS and WRKY transcription factors	Gomphrenin-I, Phyllocactin-II, Isophyllocactin, L-tyrosine, Cyanidin 3-O-galactoside, Cyanidin -O-glucoside-O-rhamnoside, amaranthine, Petunidin, Betanin, malvidin 3-o- galactoside and oenin chloride	[137]

**Table 4:** Reference genomes of tropical fruits.

Crop	Reference	Sequencing Technology Used	Percent of Assembly in Pseudo Chromosomes	Assembly Level	Estimated Genome Assembly size	Public Availability (NCBI ID)
Mango (Cultivar ‘Amrapalli’)	[234]	SMRT sequencing	73.2	Chromosome	323 Mb	Not available
Mango (Cultivar ‘Alphonso’)	[235]	SMRT sequencing	91.1	Chromosome	392.9 Mb	CATAS_Mindica_2.1 GenBank accession: GCA_011075055.1
Mango (Cultivar ‘Hong Xiang Ya’)	[97]	SMRT sequencing	98.7	Chromosome	389 Mb	Not available
Mango (Cultivar ‘Tommy Atkins’)	[236]	Illumina HiSeq2500	89	Chromosome	377 Mb	ASM1674641v1 GenBank accession: GCA_016746415.1
Banana- (*Musa acuminata*)	[237]	Sanger and Roche/454 (GSFLX pyrosequencing)	87.8	Contig	523 Mb	DDBJ/EMBL/ GenBank accession numbers CAIC01000001–CAIC01024424 (contigs)
Banana (*Musa balbisiana*)	[238]	Illumina HiSeq 2000 II	78.9	Contig	402.5 Mb.	GenBank SAMN02333823
Banana (*Musa itinerans*)	[239]	Illumina Hiseq2000	75.2	Contigs	462.1 Mb	PRJNA312694
Citrus (*Fortunella hindsii*)	[240]	PacBio Sequel, 10×Genomics Chromium	96.9	Contig	373.6 Mb	ASM480246v1 PRJNA487160
Citrus (*Citrus unshiu*)	[241]	Illumina HiSeq 2000	94.2	Contig	359.2 Mb	CUMW_v1.0 PRJDB5882
Citrus (*Citrus clementina*)	[242]	Long-read 454 and Sanger expressed-sequence-tags (ESTs)	97	Scaffold	301.3 Mb	PRJNA232045, PRJNA223006
Citrus (*Citrus reticulata*)	[243]	Illumina shotgun sequencing	90	Scaffold	344.2 Mb	PRJNA388397, INSDC: NIHA00000000.1
Citrus (*Citrus medica*)	[244]	PacBio RS II platform via a shotgun approach	87	Scaffold	406 Mb	PRJNA320023
Papaya (Cultivar ‘SunUp’)	[245]	Whole-genome shotgun (WGS) sequencing	74.2	contigs	372 Mb	GenBank under accession number ABIM00000000.
Guava (Cultivar ‘New Age’)	[231]	SMRT sequencing	95.7	Chromosome	463.8 Mb	guava_v11.23 GenBank assembly accession: GCA_016432845.1
Jack fruit (*A. heterophyllus*)	[233]	IIlumina HiSeq 2000	98.98	Chromosome	1.2 Gb	Not available
Dragon fruit (Cultivar ‘Guanhuabai’)	[74]	PacBio, Illumina, 10× Genomics, and Hi-C,	97.67	Chromosome	1.41 Gb	Not available
Dragon fruit (Cultivar ‘David Bowie’)	[116]	10×chromium sequencing, Hi-C	88.7	Chromosome	1.33 Gb	ASM1758966v1 GenBank assembly accession: GCA_017589665.1
Durian (*Durio ziberthinus*)	[246]	PacBio, Hi-C	96.88	Chromosome	738 Mb	Duzib1.0 GenBank assembly accession: GCA_002303985.1

**Table 5:** Application of genetic engineering for fruit trait improvement.

Fruit Crop	Targeted Genes/Pathways	Genetic Engineering Approach	Modified Traits	Reference
**Apple**: Arctic^®^Golden Delicious, Arctic^®^ Granny Smith, and Arctic^®^ Fuji	Polyphenoloxidases- *PPO2*, *GPO3*, *APO5* and *pSR7*	RNAi technology	Non-browning	[307]
**Pineapple** (Pinkglow™)	Carotenoid pathway- tangerine (*Citrus reticulata*) *PSY* gene	Transgenic	Pink flesh	[308]
**Squash**	Coat protein gene transfer	Transgenic	Resistant to potyviruses	[309]
**Banana**	PSY and Ferritin gene	Transgenic	Biofortifed pro-vitamin A and Iron	[299,300]
**Grand Naine banana**	Endochitinase gene *ThEn-42* (*Trichoderma harzianum)*, grape stilbene synthase (*StSy*), and superoxide dismutase Cu, *Zn-SOD* was taken from tomato	Transgenic	Improved tolerance toward fungal disease	[301]
**Banana**	*MaMADS*, MaMADS36	RNAi technology	Delayed ripening	[310]
**Cavendish banana**	Banana cisgenes	Cisgenic	Enhanced level of provitamin-A and to increase the resistance to Panama disease	[298]
**Banana**	*MaACO1* (amino-cyclopropane-1-carboxylate oxidase 1)	CRISPR/Cas9	Reduced ethylene synthesis and extended shelf life	[311]
**Cavendish banana**	Lycopene epsilon-cyclase (*LCY ε*) gene	CRISPR/Cas9	β-carotene enrichment	[312]
**Papaya**	Coat protein (CP) mediated	RNAi technology	PRSV-resistant	[304]
**Papaya**	S-genes	CRISPR/Cas9	Resistance to PRSV	[313]
**Duncan grapefruit**	*CsLOB1*	CRISPR/Cas9-mediated gene knockouts	Duncan grapefruit canker	[314]
**Sweet orange**	Silencing of β-carotene hydroxylase	RNAi technology	Accumulation of carotenoids in fruit pulp	[315]

**Table 6:** Crop specific databases in tropical fruits.

Crop	Database	Developed Using	Purpose	Reference	Weblink (Accessed on 10 September 2021)
Banana	MusatransSSRDB	Transcriptome	selection of SSR primers for a specific objective	[328]	http://nrcb.res.in/nrcbbio/about.html
	Musa Germplasm Information System (MGIS)	Accession-based data and genotyping studies	global ex situ-held banana genetic resources	[330]	https://www.crop-diversity.org/mgis/
	Banana Genome Hub	Genomic data on banana	integration between various systems (Jbrowse, Galaxy, Gigwa	[331]	banana-genome-hub. southgreen.fr
	BanSatDB	Whole genome-based	(>341,000) of putative STR markers from *Musa* genera and 580 validated STR markers from the published literature	[332]	http://webtom.cabgrid.res.in/bansatdb/
Mango	MGdb	Transcriptome	Genomic resource	[333]	—
	Mango Bienniality Gene Database (MBGDB)	NCBI	Genes related to bienniality rhythm of mango	[334]	http://webapp.cabgrid.res.in/mangodb
	MiSNPDb	Sequence based	Phylogenetic and evolutionary studies using SNPs	[335]	http://webtom.cabgrid.res.in/mangosnps/
Citrus	CitGVD	Published resources	Citrus genomic variation database	[336]	http://citgvd.cric.cn/home
	Citrus genome database (CGD)	Genomics, genetics and breeding	Genomics, genetics and breeding and disease resistance	—	https://www.citrusgenomedb.org/
	iCitrus	NCBI	Citrus protein identification	[329]	—
Papaya	Carica papaya Genome DB—PlantGDB	Whole genome	Comparative plant genomics	[337]	http://www.plantgdb.org/CpGDB/
Durian	MaGenDB	Genome information from public databases	Genomic resource for Malvaceae family	[338]	http://magen.whu.edu.cn

## Data Availability

Available with the manuscript.
